# Effects of M/G Ratios of Sodium Alginate on Physicochemical Stability and Calcium Release Behavior of Pickering Emulsion Stabilized by Calcium Carbonate

**DOI:** 10.3389/fnut.2021.818290

**Published:** 2022-01-11

**Authors:** Xiaotong Yang, Haomin Sui, Hongshan Liang, Jing Li, Bin Li

**Affiliations:** College of Food Science and Technology, Huazhong Agricultural University, Wuhan, China

**Keywords:** calcium carbonate, sodium alginate, Pickering emulsion, calcium supplement, stability, rheological properties, M/G ratio, release characteristics

## Abstract

The gel properties of sodium alginate (SA) have been revealed to be strongly correlated with its ratio of D-mannuronate to L-guluronate (M/G ratio). Herein, we focused on SA with different M/G ratios to conduct an in-depth study on the effect of the M/G ratio difference on physicochemical stability and calcium release behavior of the Pickering emulsion stabilized by calcium carbonate (CaCO_3_). The oil phase was added to the aqueous phase, prepared by SA with different M/G ratios (2.23, 0.89, and 0.56) and CaCO_3_, for one-step shearing to obtain the E1, E2, and E3 emulsions, respectively. The results of the particle size, microstructure, long-term stability, rheological, and microrheological properties of the emulsions showed that the E3 emulsion, prepared by SA with a smaller M/G ratio, had a smaller particle size and has remained in a flow condition during the long-term storage, while the E1 and E2 emulsions had a gelation behavior and a stronger viscoelasticity. Moreover, the emulsion, as a liquid calcium supplement, is not only convenient for oral intake while meeting the calcium needs of the body, but also controls the release of Ca^2+^. The calcium release of the emulsions in a simulated gastric environment demonstrated that the calcium release ratio increased with the decrease of SA concentration, with the increase of M/G ratio, and with the decrease of oil phase volume.

## Introduction

Calcium is an essential nutrient in the human body, thus, playing a critical role in maintaining various physiological functions, as well as strengthening the bone structure (1). Research indicated that the average daily calcium intake in many countries, including China, was less than 400 mg, which did not meet the recommended intake (2). Importantly, calcium deficiency often leads to diseases such as osteoporosis, hypertension, and colorectal cancer (3). Therefore, calcium supplements are often used to meet the needs of the body due to the low absorption rate of calcium in the diet. Calcium carbonate, the most common form of calcium supplements, shows low bioavailability (4, 5). Hence, a sustainable and cost-effective strategy to improve calcium carriers to increase the absorption ratio of calcium has become the industry demand (6).

Pickering emulsions have a good stability, biosafety, and biocompatibility (7, 8), and are widely used in the field of nutraceutical carriers, which can be used for controlling release and for effectively improving the stability, delivery efficiency, and bio-accessibility of nutrients (9–13). Thus, Pickering emulsion, stabilized by CaCO_3_ as calcium supplement, has a great potential to satisfy the factory demands. Nevertheless, due to the small size of CaCO_3_ particles with strong surface energy, it was always easy to agglomerate and hard to form a stable emulsion (14). Hence, we added SA to the emulsion and changed the preparation method, thus, innovatively discovered that the CaCO_3_/SA emulsion prepared by a one-step emulsifying method could maintain the liquid properties for a long time, which makes it promising for the preparations that are efficiently loaded with calcium.

Additionally, α-L-guluronate (G blocks) in sodium alginate was crosslinked with Ca^2+^ to form a gel structure (15), and the difference of M/G ratios had certain influence on the functional properties of SA (16–18). Therefore, we hypothesized that the difference of M/G ratio in SA would not only affect the stability of CaCO_3_/SA emulsion system but also significantly interfere with the release of calcium. Herein, under the condition that the molecular weight was basically similar, three kinds of SA with different M/G ratios (2.23, 0.89, and 0.56) were selected to investigate their influence on the stability and the rheological properties of the emulsion. At the same time, the difference of calcium release caused by M/G ratio difference of SA was further explored. This study is expected to provide new ideas and data support for the preparation of emulsion-based calcium supplement and the adjustment of calcium release characteristics.

## Materials and Methods

### Materials

Soybean oil was obtained from Yihai Kerry Co., Ltd. (Shenzhen, China). Heavy CaCO_3_ was obtained from Zhengzhou Ruipu Bioengineering Co., Ltd. (Henan, China). Sodium alginate was obtained from Qingdao Haizhilin Biological Co., Ltd. (Shandong, China). Pepsin was purchased from Shanghai yuanye Bio-Technology Co., Ltd. (China).

### Determination of Structure and Properties of SA

#### Structure

The three kinds of SA extracted from *Laminaria japonica, Macrocystis pyrifera*, and *Lessonia trabeculata* were named SA-1, SA-2, and SA-3, respectively. Accurately weighed SA-1, SA-2, and SA-3 were dissolved in ultrapure water to obtain 0.5% (w/w) SA-1, SA-2, and SA-3 solution, and then the pH of the solution was adjusted to 3. After boiling the water bath for 30 min, we adjusted the pH to 7 (19). The SA-1, SA-2, and SA-3 solution was freeze-dried for further measurement. The molecular structures of SA-1, SA-2, and SA-3 were determined by the electron paramagnetic resonance spectroscopy (MS5000XM5000X, MagnettechGmbH, Germany). Accurately weighed samples were placed in a nuclear magnetic tube and were dissolved in D_2_O. The M/G ratio of SA-1, SA-2, and SA-3 was analyzed by ^1^H NMR; the measurement parameter was 600 MHz.

#### Molecular Weight

With some modifications, the molecular weight of SA was measured according to the method described by Li et al. (20). Accurately weighed SA-1, SA-2, and SA-3 were dissolved in 0.1 mol/L NaNO_3_ solution containing 0.02% NaN_3_ to obtain 4 mg/mL SA-1, SA-2, and SA-3 solution. The molecular weight of the sample was determined by a laser light scattering spectrometer (DAWM HELEOSII, Wyatt Technology Co., Ltd., USA). The SB-805 HQ gel column was selected, then adjusted the column temperature to 30°C. The mobile phase was 0.1 mol/L NaNO_3_ solution containing 0.02% NaN_3_, the flow velocity was 0.4 mL/min, the sample size was 100 μl, and the retention time was 60 min.

#### Viscosity

Respectively, the SA-1, SA-2, and SA-3 (5 g) were dissolved in an ultrapure water (500 mL), and then the sample solutions were placed in a refrigerator at 4°C until the bubbles were removed. The viscosity of the SA-1, SA-2, and SA-3 solutions was measured with a rotary viscometer (NDJ-8S, Shanghai Precision Instrument Co., Ltd., China), and the rotating speed was set at 30 r/min, 30 r/min, and 12 r/min, respectively (21). The rotation time was 30 s, and the solution temperature was 20 ± 0.5°C. After the value was stabilized, the reading was the viscosity number.

### Preparation of Pickering Emulsion

#### Preparation of CaCO_3_ Particles

The food grade-heavy CaCO_3_ was dried in an oven (60°C) and was ground by a high-energy nano ball mill for 8 h (CJM-SY-B, Qinhuangdao Taiji Ring Nano-Products Co., Ltd., Hebei, China). The CaCO_3_ particles with small particle size were obtained and were named CaCO_3_-8.

#### Pickering Emulsions With Sodium Alginate of Different M/G Ratios

The SA-1 powders (0.2, 0.25, and 0.3 g) were dissolved in an ultrapure water (20 g), and were dispersed by using a magnetic stirrer (SP-300, Hangzhou Miu Instrument Co., Ltd., Zhejiang, China) at 550 r/min for 1.5 h to prepare the SA-1 solutions (1, 1.25, and 1.5 wt%). Subsequently, it was stored overnight in a refrigerator (4°C). The CaCO_3_-8 (1.7 g) was dispersed in SA-1 solutions to obtain an aqueous phase (8.5wt % CaCO_3_) by a magnetic stirrer at 550 r/min for 0.5 h. The soybean oil (4 g) was also added to the aqueous phase and was sheared by a high-speed shearing machine (Ultra-Turrax T25, IKA Works, Guangzhou, China) at 12,000 r/min for 6 min to obtain the emulsion stabilized by CaCO_3_ and SA-1. The sodium azide (0.02%) was added to the emulsion to inhibit microbial growth (22) and was stored at room temperature (25°C) for the subsequent measurements. The Pickering emulsion obtained by this method was named E1. In the same way, Pickering emulsions prepared by SA-2 and SA-3 were named E2 and E3, respectively.

#### Pickering Emulsions With Different Water-Oil Ratios

The SA-3 was used as raw material. The preparation method, as described in Section Pickering Emulsions With Sodium Alginate of Different M/G Ratios, was adopted to change the added amount of the soybean oil, while the other preparation conditions remained unchanged. Pickering emulsions with water-oil ratios of 20:1 and 10:1 were obtained by adding 1 and 2 g soybean oil to the water phase (20 g) for shearing, and were named O1 and O2, respectively. The emulsions O3 and E3 were the same sample in the subsequent experiments as they were prepared with the same water-oil ratio and sodium alginate (SA-3).

### Determination of Particle Size of Emulsion

A laser particle size analyzer (Mastersizer 2000, British Malvern Instrument Co., Ltd., UK) was used to measure the particle size. Water (refractive index: 1.333) was set as dispersant, soybean oil (refractive index: 1.475) was set as sample material, laser shading index ranged from 1 to 20%, and pump speed was 2,000 r/min. The measurement results were represented by the volume mean diameter (D[4, 3]) (23) with the following formula:


$$\begin{array}{l} \text{D}\left[4,3\right]={\sum \text{n}}_{\text{i}}{\text{d}}_{\text{i}}^{4}\;/{\sum \text{n}}_{\text{i}}{\text{d}}_{\text{i}}^{3} \end{array}$$

where *d*_*i*_ was the droplet diameter of emulsion (μm) and *n*_*i*_ was the number of emulsion droplets with a particle size of *d*_*i*_.

### Determination of Creaming Index

The height of the supernatant layer and emulsified layer were recorded for 0, 1, 2, 7, 14, and 28 days. The degree of emulsification of emulsion was represented by the creaming index (CI) (24) with the following formula:


$$\begin{array}{l} \text{CI}\left(\text{\%}\right)={\text{H}}_{\text{s}}/{\text{H}}_{\text{t}}\times 100 \end{array}$$

where *H*_*s*_ was the height of the supernatant layer of the emulsion (cm) and *H*_*t*_ was the height of the emulsion (cm).

### Observation of Micromorphology

The microstructure of emulsion was observed by optical microscope (OD1400Y, Ningbo Sunny Instruments Co., Ltd., Zhejiang, China) under 20 times objective lens.

### Determination of Rheological Properties

A rheometer (DHR2, TA Instrument Co., Ltd., USA) was used to measure the rheological properties of emulsions with varying storage time. An aluminum parallel plate with a diameter of 60 mm and 500 μm gap was used for measuring the fixture. The dynamic frequency sweep test was measured in the frequency range of 0.1–100 rad/s with 0.06% strain. The steady-state shear sweep test was carried out at a shear rate of 0.01–100 s^−1^ and at a fixed frequency of 1 Hz. All measurements were measured at 25°C (25).

### Determination of Microrheological Properties

An optical micro-rheometer (Rheolaser Master, Formulaction Instrument Co., Ltd., France) was used to obtain the mean square displacement (MSD) of the emulsion. The sample information was obtained by scanning every 150 s at 25°C. Subsequently, the RheoSoft Master software was used to calculate the solid-liquid equilibrium balance (SLB) values, elasticity index (EI), and the macroscopic viscosity index (MVI) (26).

### *In vitro* Digestion of Emulsion

#### In vitro Digestion Experiment

Simulated gastric fluid (SGF) containing NaCl (2 mg/ml) and pepsin (3.2 mg/ml), with the pH adjusted to 1.5 using 1.0 M HCl, was prepared (27). The emulsion (5 g) was added into the SGF (100 ml), and it was incubated and oscillated in a water bath at 120 r/min for 12 h at 37°C. The mixed solution (100 μl) was taken out every 1 h and measured the release of calcium during digestion. At the same time, the same volume of SGF was added, so that the solution volume remains unchanged, while the pH was controlled within the range of 1–2.

#### Determination of Calcium Release Ratio

The calcium content in emulsion was measured according to the method described by Udoh et al. (28) with some modifications. Standard solutions with calcium concentration of 0, 3, 6, 9, 12, 15, and 18 μg/ml were, respectively, prepared. The absorbance was measured by atomic absorption spectrophotometer (AA-6300C, Shimadzu Instruments Manufacturing Co., Ltd., Japan) at 422.7 nm, and the standard curve was drawn. The linear regression equation was *y* = 0.0651x + 0.062. The correlation coefficient (R^2^) was 0.9996.

The sample was diluted with 1% nitric acid and was filtered through a 0.45 μm filter membrane to obtain the filtrate. Then, the 9.5 ml filtrate and the 0.5 ml lanthanum solution were mixed to obtain the solution to be tested. The absorbance of the solution to be tested was likewise measured at 422.7 nm, and its calcium concentration was obtained by referring to the standard curve. The calculation formula of the calcium release ratio was as follows:


$$\begin{array}{l} \text{Calcium release ratio }\left(\text{\%}\right)=\text{m}/{\text{m}}_{0}\times 100 \end{array}$$

where *m* was the calcium concentration in the mixed solution (μg/ml), and *m*_0_ was the calcium concentration in the emulsion used for digestion (μg/ml).

### Data Analysis

Each experiment was repeated three times with at least three parallels for each measurement. The measurement result was the mean ± standard deviation and *P* < 0.05 was considered statistically significant. All statistical analyses were performed by SPSS software (SPSS version 25.0, IBM Institute, USA).

## Results and Discussion

### Structure and Properties of Sodium Alginate

To explore the influence of the M/G ratio of SA on the properties of emulsion, an SA with a different M/G ratio was selected for the experiment. However, in addition to the M/G ratio, its molecular weight and viscosity were also the important factors affecting the properties of SA, which would affect the properties of the emulsion. Therefore, the physicochemical properties of SA-1, SA-2, and SA-3 were determined.

To obtain the exact value of M/G ratio of SA, the ^1^H NMR spectra was analyzed. The molar fractions of the monads (F_G_ and F_M_) and the M/G ratio were calculated employing the formula given by Grasdalen et al. (29). The F_M_ and F_G_ of SA-1, SA-2, and SA-3 were 0.69 and 0.31, 0.47 and 0.53, and 0.36 and 0.64, respectively. Subsequently the M/G ratios of SA-1, SA-2, and SA-3 were 2.23, 0.89, and 0.56 by further calculation (Table 1). The molecular weight and distribution of SA-1, SA-2, and SA-3 were determined by gel permeation chromatography. As shown in Table 1, the weight-average molecular weight (M_W_) of SA-1, SA-2, and SA-3 were 1.799×10^5^, 1.772×10^5^, and 1.795×10^5^, respectively, while the number-average molecular weight (M_N_) was 1.419×10^5^, 1.520×10^5^, and 1.226×10^5^, respctively, thus, showing no significant difference. Moreover, the viscosity of SA-1, SA-2, and SA-3 also showed no significant difference.

**Table 1:** Results of determination of physical and chemical properties of SA.

**SA type**	**M_**W**_**	**M_**N**_**	**M_**W**_/M_**N**_**	**M/G ratio**	**Viscosity (mPa·s)**
SA-1	1.799 × 10^5^	1.419 × 10^5^	1.268	2.23	155.8
SA-2	1.772 × 10^5^	1.520 × 10^5^	1.166	0.89	170.6
SA-3	1.795 × 10^5^	1.226 × 10^5^	1.464	0.56	125.5

To summarize, the M/G ratios of SA-1, SA-2, and SA-3 were significantly different from high to low, while there were no significant differences in molecular weight and viscosity, which met the experimental requirements.

### Effect of Sodium Alginate of Different M/G Ratios on Properties of Emulsion

#### Stability

**Figure 1:**
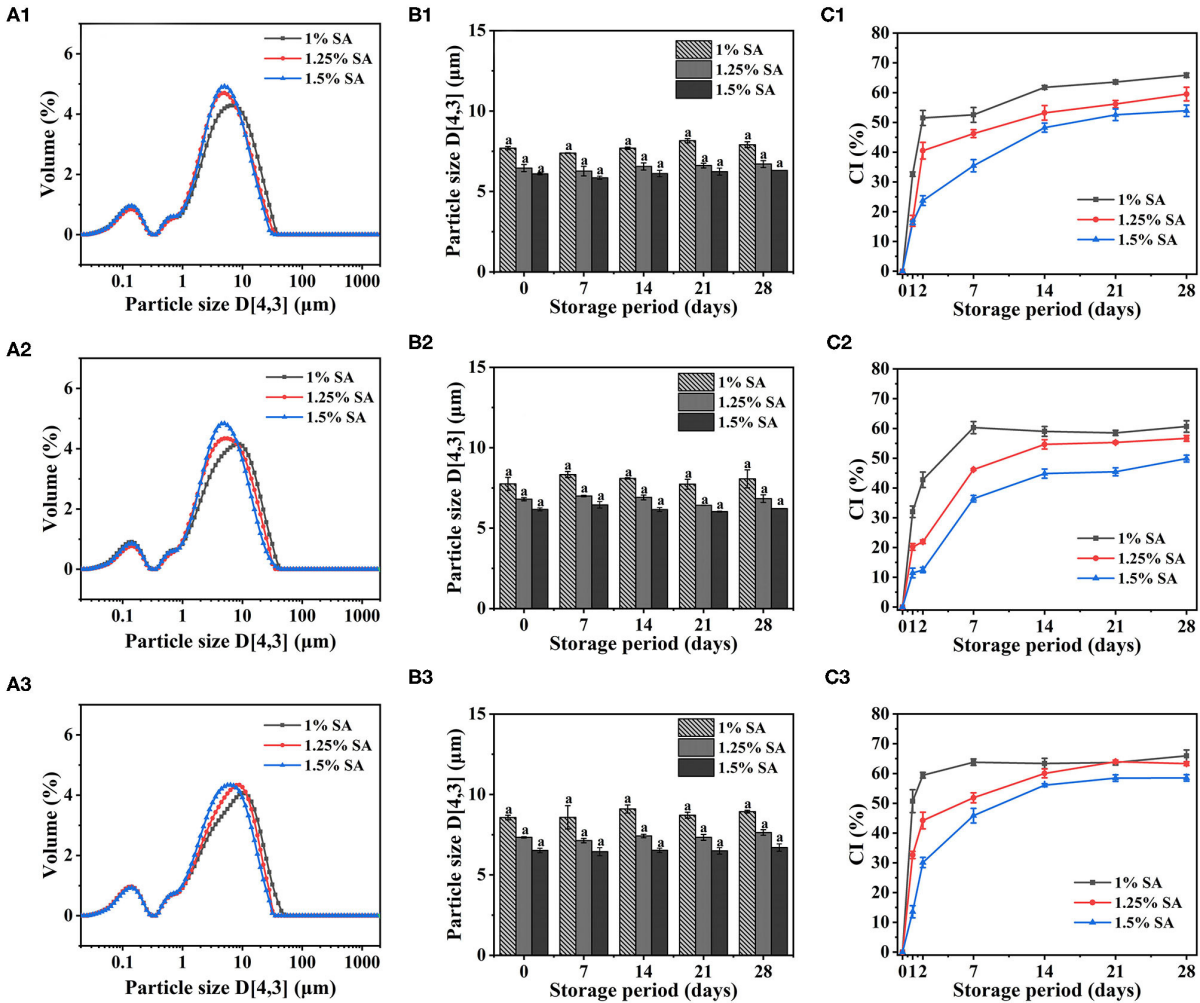
Particle size distribution of E1 **(A1)**, E2 **(A2)**, and E3 **(A3)** emulsions. Particle size and creaming index of E1 **(B1,C1)**, E2 **(B2,C2)**, and E3 **(B3,C3)** emulsions at different storage times. The error bars represent standard errors of the means.

**Figure 2:**
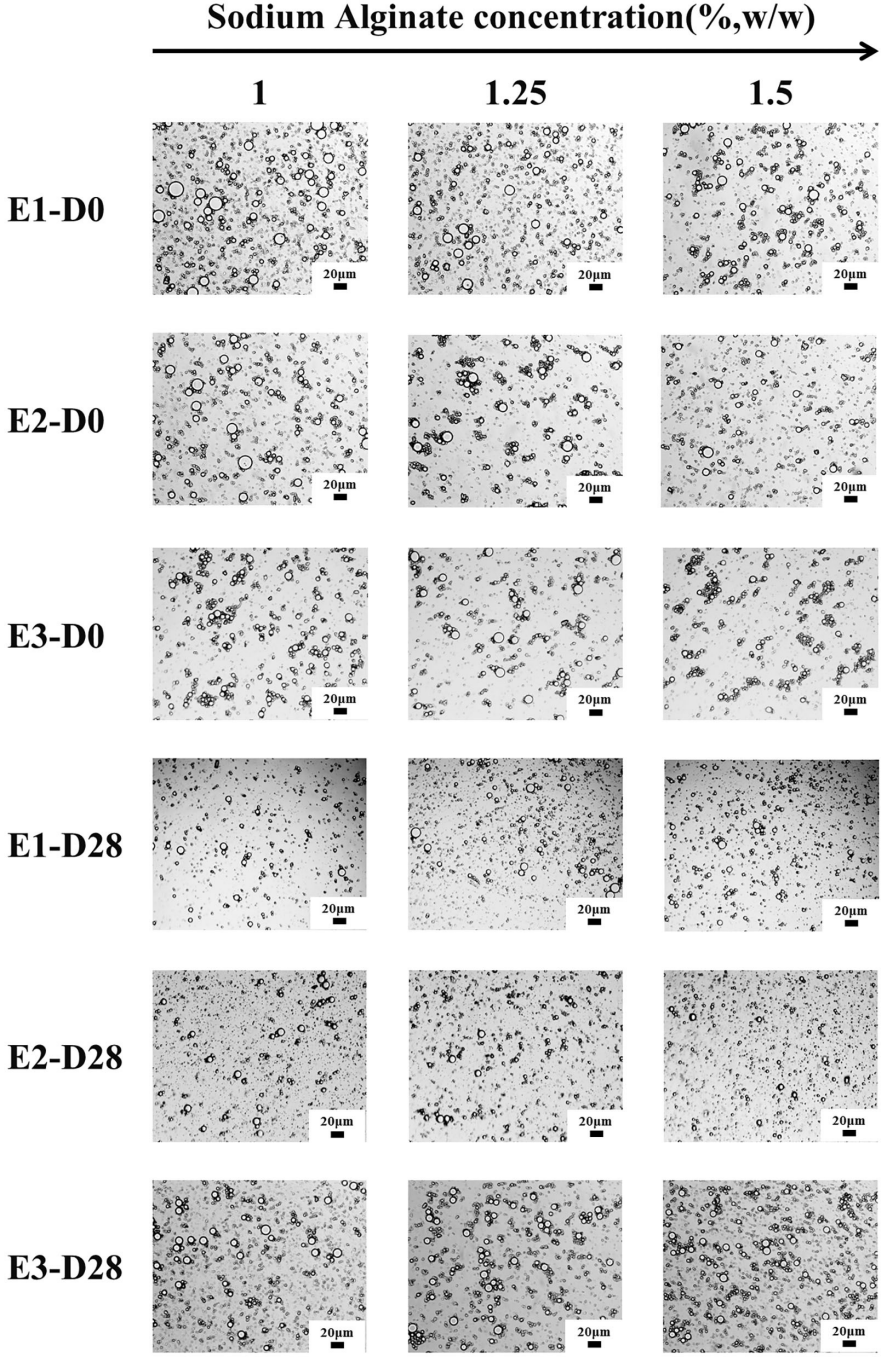
Optical microstructure images of E1–E3 emulsions at different storage times (scale bar is 20 μm). D0 and D28 represent emulsions stored for 0 and 28 days.

The average particle size of E1, E2, and E3 emulsion was about 5-9 μm, and the particle size did not significantly change within 28 days of storage (Figures 1B1–B3). The particle size of the emulsions observed under a 20-fold objective lens was consistent with that of the measured results. Moreover, the microstructure of E1, E2, and E3 emulsions was not damaged after 28 days of storage, and they all showed structural characteristics similar to that of fresh emulsions (Figure 2). These phenomena indicated that E1, E2, and E3 emulsions had good stability during 28 days of storage. As can be seen from the emulsion particle size distribution (Figures 1A1–A3), with the increase of the SA concentration, the emulsion particle size decreased, and the distribution peak width became narrower. The results indicated that the increase of SA concentration could cause smaller droplet size and a more uniform particle size distribution to the emulsion, which was beneficial to improve the stability of the emulsion. Yan et al. (30) also found that the particle size of emulsion gradually decreased with the increase of emulsifier concentration.

In addition, CI of E1, E2, and E3 emulsion increased rapidly at 0-2 days and reached equilibrium at 7-14 days. There was no significant difference in CI of E1, E2, and E3 emulsions on day 28, and CI value decreased with the increase of SA concentration, revealing that the stability of emulsions has enhanced (Figures 1C1–C3). This was due to the fact that with the increase of SA concentration, more particles were provided for droplet formation, which contributed to the formation of a firm interface layer on the oil-water interface, thus, helping to weaken droplet aggregation and thereby improving the stability of the emulsion. Wang et al. (31) made similar findings in the study of casein gel particle stabilized Pickering emulsions.

#### Macro-Morphology

**Figure 3:**
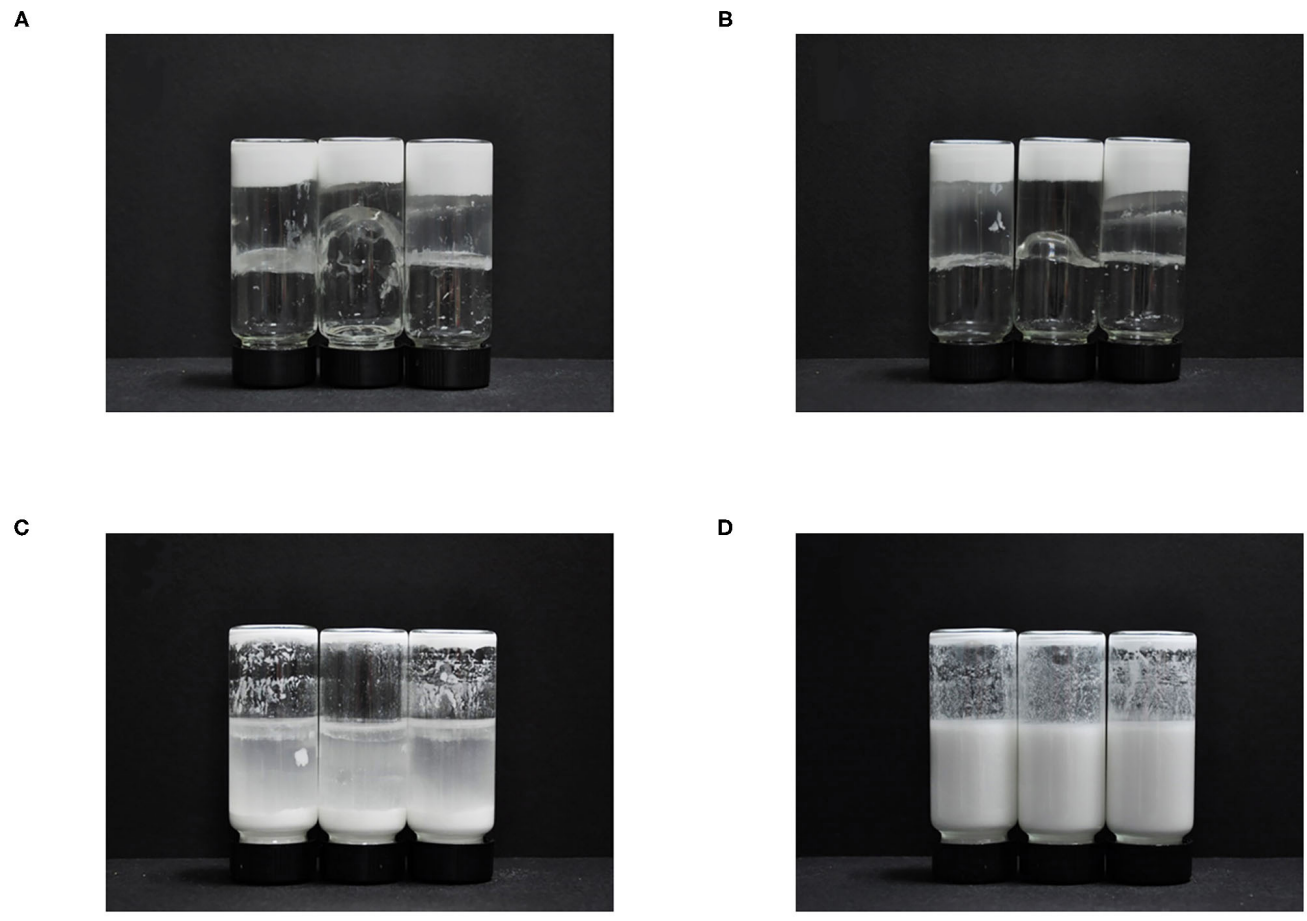
Photos of CaCO_3_/SA emulsions after 6 months of storage: E1 **(A)**, E2 **(B)**, and E3 **(C)** emulsions stored at rest and E3 emulsion stored at rest for 20 min after vigorous shaking **(D)**.

To investigate the effect of SA with different M/G ratios on the long-term stability of emulsions, the appearance of E1, E2, and E3 emulsions stored for 6 months were photographed and recorded, as shown in Figure 3. The E1, E2 and E3 emulsions showed obvious differences in structure characteristics. The E1 and E2 emulsions showed gelation behavior, while the E3 emulsions has always maintained liquid during storage. Research has shown that the G blocks in SA have chelated with the Ca^2+^ to form a similar “egg box” structure (32). This “egg box” structure was bound horizontally and formed a stable three-dimensional network structure, which promoted the formation of gel structure. Therefore, the reason for gelation of E1 and E2 emulsions was that there were few G blocks binding the Ca^2+^, and a small part of Ca^2+^ could complex with these binding sites to form a gel structure. However, the E3 emulsion required more Ca^2+^ to be crosslinked with SA to achieve the gel state, so the E3 emulsion could maintain the flow state for a longer time.

#### Rheological Properties

**Figure 4:**
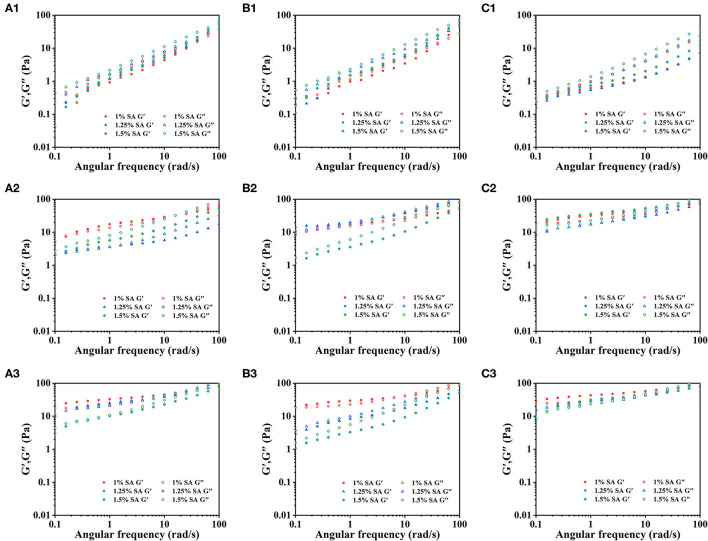
Dynamic frequency sweep images: E1 emulsions stored for 0 **(A1)**, 14 **(A2)**, and 28 **(A3)** days; E2 emulsions stored for 0 **(B1)**, 14 **(B2)**, and 28 **(B3)** days; E3 emulsions stored for 0 **(C1)**, 14 **(C2)**, and 28 **(C3)** days.

To further analyze the relationship between the formation of gel network structure of emulsion and SA during storage, the rheological properties of E1, E2, and E3 emulsions were measured. Figure 4 showed the dynamic frequency sweep images of E1, E2, and E3 emulsions on day 0, 14 and 28. The elastic modulus (G') of E1 and E2' fresh emulsions was less than the viscous modulus (G”) at the stage of initial angular frequency increase. E1 and E2 fresh emulsions exhibited liquid properties, and their viscous behavior was dominated. Moreover, the G' of E1 and E2 emulsions was increased with the increase of angular frequency, and the G' was greater than G” when the angular frequency was larger, hinting that a weak gel-like structure was formed. However, G' of E3 emulsion was always less than G”, demonstrating that its viscous behavior was dominant and was mainly manifested as the liquid characteristics. The G' and G” of E1, E2, and E3 emulsion increased with the storage time, indicating that a more stable three-dimensional network structure was formed. It might be due to the chelation between the Ca^2+^ and the G blocks in SA, which developed the gel structure (32). Besides, G' and G” of E3 emulsion stored for 14 and 28 days were greater than E1 and E2 emulsions, revealing that the gel structure of E3 emulsion was stronger.

**Figure 5:**
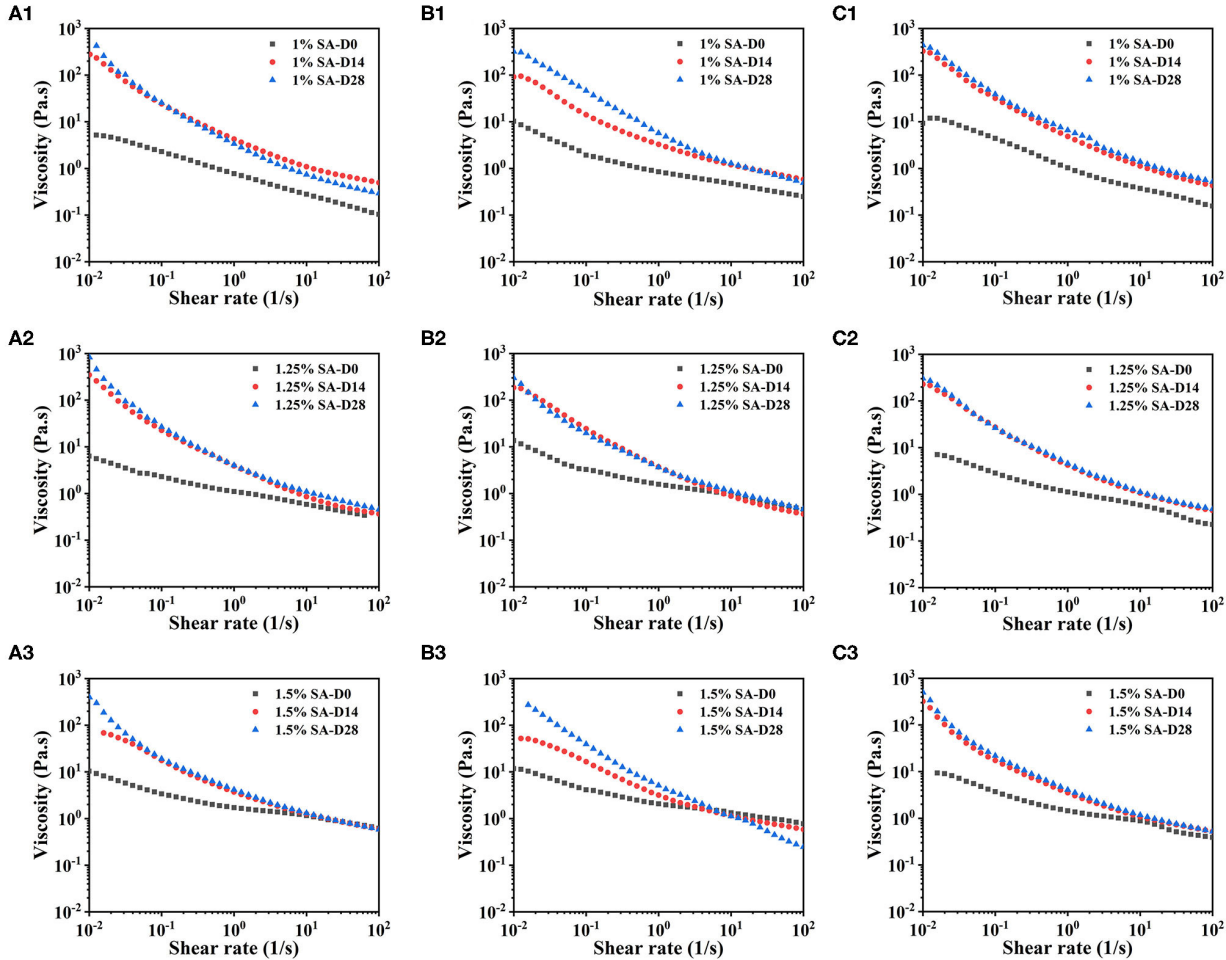
Steady-state shear sweep images: E1 emulsion with 1% SA **(A1)**, 1.25% SA **(A2)**, and 1.5% SA **(A3)**, E2 emulsion with 1% SA **(B1)**, 1.25% SA **(B2)**, and 1.5% SA **(B3)**, E3 emulsion with 1% SA **(C1)**, 1.25% SA **(C2)**, and 1.5% SA **(C3)**. D0, D14 and D28 represent emulsions stored for 0, 14 and 28 days.

Figure 5 showed the steady-state shear sweep images of E1, E2, and E3 emulsions on day 0, 14, and 28. The viscosity of E1, E2, and E3 emulsions decreased with the increase of the shear rate, showing typical non-Newtonian fluid characteristics. This mainly reflected the deflocculation between emulsion droplets (33, 34). With the increase of storage time, the E1, E2, and E3 emulsions had higher viscosity and more obvious shear thinning behavior, which might be due to the three-dimensional network that was developed in the emulsion during storage.

#### Microrheological Properties

The determination of microrheological properties of E1, E2, and E3 emulsions was also helpful to understand the gelation behavior of emulsions during the long-term storage. The micro-rheology method was a non-destructive technique, which used the Brownian motion principle of particles to measure. The measurement process did not damage the sample structure and could be used to obtain information for long-term storage system (35).

**Figure 6:**
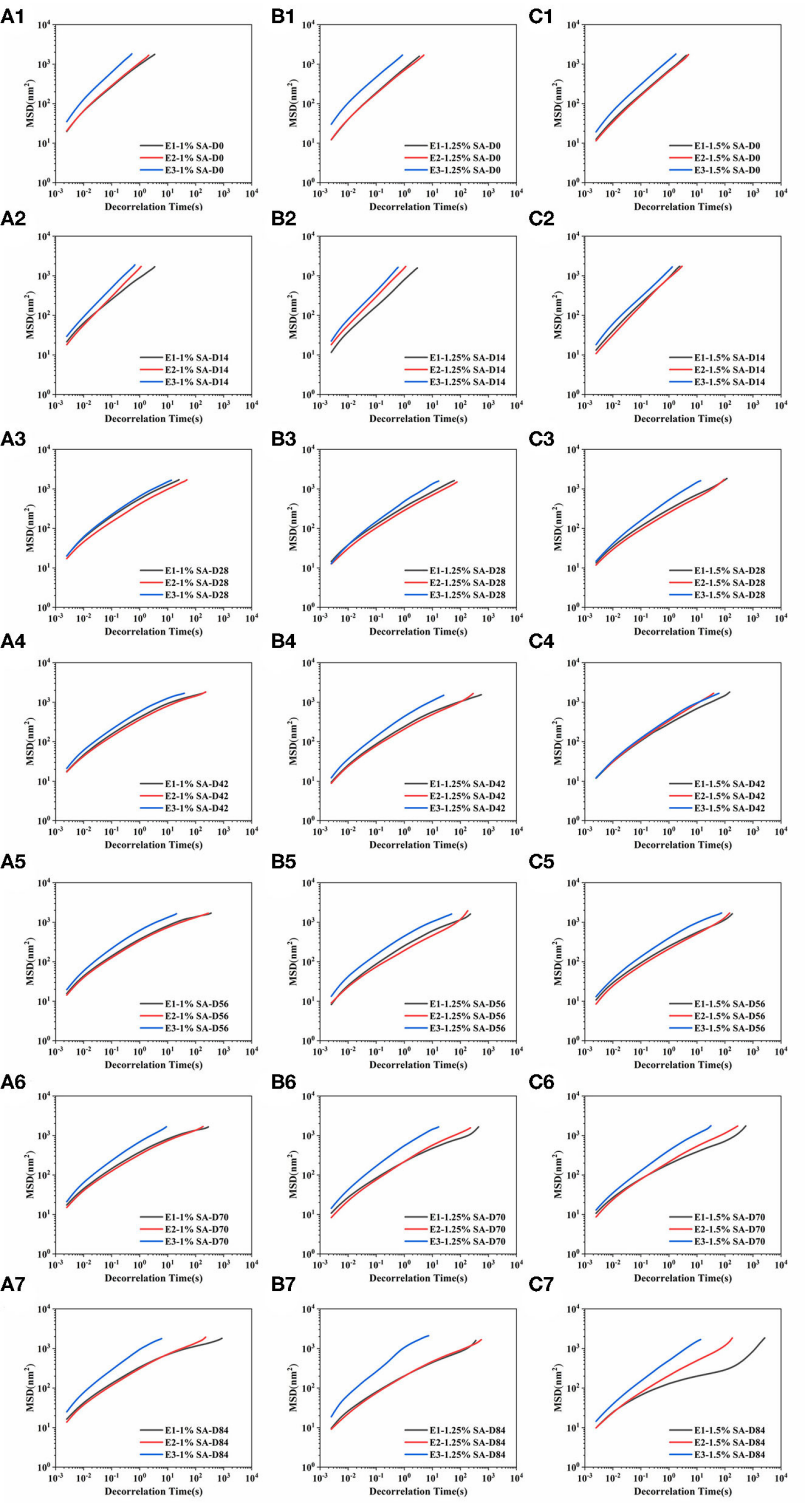
Mean squared displacement curve: E1 emulsion stored for 0 **(A1)**, 14 **(A2)**, 28 **(A3)**, 42 **(A4)**,56 **(A5)**, 70 **(A6)**, and 84 **(A7)** days, E2 emulsion stored for 0 **(B1)**, 14 **(B2)**, 28 **(B3)**, 42 **(B4)**,56 **(B5)**, 70 **(B6)**, and 84 **(B7)** days, E3 emulsion stored for 0 **(C1)**, 14 **(C2)**, 28 **(C3)**, 42 **(C4)**, 56 **(C5)**, 70 **(C6)**, and 84 **(C7)** days. D0, D14, D28, D42, D56, D70, and D84 represent emulsions stored for 0, 14, 28, 42, 56, 70, and 84 days.

The MSD represented the mean area of particles movement in the sample during a given decorrelation time. The MSD curves of E1, E2, and E3 emulsions on days 0, 14, 28, 42, 56, 70, and 84 were shown in Figure 6. The MSD curves of E1, E2, and E3 fresh emulsions rose linearly with time, representing that the emulsions particles moved freely in the continuous phase and showed viscous characteristics. In addition, the slope of MSD curve of E1 and E2 emulsions stored for 28 and 42 days showed an uneven phenomenon, and the MSD was non-linearly correlated with the decorrelation time. These results illustrated that the movement of emulsion droplets in the emulsion system was limited, and there was a network structure that trapped the droplet movement (36). The linear characteristics of MSD curves of E1 and E2 emulsions on day 56 and 84 gradually weakened and a relaxation platform appeared, demonstrating that particles were trapped in the three-dimensional microstructure network. Compared with E3 emulsions, E1 and E2 emulsions had smaller slopes and longer decorrelation time of MSD curve, proving that the movement of E1 and E2 emulsion droplets was strongly restricted, and the viscosity of the emulsions were higher. During this period, the slope of the MSD curve of E3 emulsion had no significant difference with the change of decorrelation time, and the slope was less than 1, representing that the droplet movement was relatively free.

Solid liquid balance (SLB) value, a ratio between the solid-like and the liquid-like behavior of the sample, is obtained from the MSD curve to reflect the solid-liquid properties of the sample. The samples are dominated by elastic and solid properties, with the SLB ranging between 0 and 0.5. The samples reached a solid-liquid equilibrium state when the SLB was 0.5, while the sample is dominated by viscous and liquid properties with the SLB ranging between 0.5 and 1 (37). As shown in Table 2, the SLB of E1, E2, and E3 emulsions stored on day 0 and 14 were between 0.5 and 1, revealing that E1, E2, and E3 emulsions are dominated by viscous behavior similar to liquids at this time. Since day 28, the SLB of E1 and E2 emulsions continuously decreased and gradually approached the solid-liquid equilibrium point. The SLB of E1 and E2 emulsions was less than 0.5 on day 84, illustrating that the three-dimensional network structure in E1 and E2 emulsions was gradually enhanced over time, while the appearance of gel network structure made the emulsions more elastic. The SLB of E3 emulsion decreased from 0.65 to 0.57 during the storage, indicating that the viscosity behavior of E3 emulsion was always dominant, and the viscosity characteristics weakened and tended to the state of solid-liquid equilibrium with the storage time. Therefore, it was speculated that the three-dimensional network structure of E3 emulsion was gradually forming, but the resulting gel structure was weak.

**Table 2:** Solid liquid balance values of E1, E2, and E3 emulsions of different storage times.

**Emulsion**	**SA**	**SLB**						
	**concentration (%)**	**D0**	**D14**	**D28**	**D42**	**D56**	**D70**	**D84**
E1	1	0.592 ± 0.025^a,G^	0.567 ± 0.013^b,E^	0.496 ± 0.003^cd,EF^	0.506 ± 0.002^c,E^	0.499 ± 0.004^cd,E^	0.463 ± 0.002^e,F^	0.484 ± 0.005^d,B^
	1.25	0.632 ± 0.019^a,CD^	0.618 ± 0.026^a,D^	0.506 ± 0.004^cd,DE^	0.519 ± 0.002^bc,D^	0.530 ± 0.002^b,C^	0.488 ± 0.006^de,E^	0.474 ± 0.003^e,B^
	1.5	0.629 ± 0.014^a,D^	0.645 ± 0.05^a,C^	0.533 ± 0.003^b,C^	0.517 ± 0.002^b,D^	0.496 ± 0.002^b,E^	0.504 ± 0.003^b,D^	0.403 ± 0.005^c,C^
E2	1	0.572 ± 0.012^b,H^	0.614 ± 0.0143^a,D^	0.508 ± 0.004^c,D^	0.492 ± 0.004^d,F^	0.488 ± 0.003^d,F^	0.489 ± 0.003^d,E^	0.490 ± 0.002^d,B^
	1.25	0.604 ± 0.011^a,F^	0.560 ± 0.009^b,E^	0.499 ± 0.007^d,DEF^	0.503 ± 0.003^d,E^	0.488 ± 0.003^e,F^	0.522 ± 0.003^c,C^	0.483 ± 0.009^e,B^
	1.5	0.615 ± 0.006^b,E^	0.718 ± 0.012^a,A^	0.491 ± 0.003^e,F^	0.548 ± 0.006^c,B^	0.502 ± 0.002^d,D^	0.506 ± 0.002^d,D^	0.502 ± 0.008^d,B^
E3	1	0.639 ± 0.018^a,BC^	0.624 ± 0.014^b,D^	0.563 ± 0.028^d,B^	0.534 ± 0.003^f,C^	0.543 ± 0.002^e,B^	0.545 ± 0.007^e,B^	0.574 ± 0.002^c,A^
	1.25	0.648 ± 0.013^b,AB^	0.665 ± 0.013^a,B^	0.614 ± 0.015^c,A^	0.555 ± 0.008^e,A^	0.548 ± 0.002^e,A^	0.549 ± 0.007^e,B^	0.573 ± 0.028^d,A^
	1.5	0.653 ± 0.008^b,A^	0.674 ± 0.006^a,B^	0.612 ± 0.002^c,A^	0.557 ± 0.003^f,A^	0.548 ± 0.002^g,A^	0.564 ± 0.003^e,A^	0.573 ± 0.002^d,A^

*D0, D14, D28, D42, D56, D70, and D84 represent different storage times of emulsions. Values with different lowercase letters represent significant difference within the line (P < 0.05). Values with different capital letters represent significant difference within the column (P < 0.05)*.

The EI represents the elastic strength of the emulsions. As shown in Table 3, the EI of fresh emulsions was small, and the elasticity of E1 and E2 emulsions was slightly stronger than E3 emulsions. The EI increased gradually with storage time, and the difference of elasticity between the E1 and E2 emulsions, as well as the E3 emulsion, was more significant. It demonstrated that the E1 and E2 emulsions can easily form a dense three-dimensional network structure with the storage time, and the time required for appearing elastic structure was shorter. In addition, E1, E2, and E3 emulsion systems showed the trend of a stronger elasticity with higher concentration of SA.

**Table 3:** Elasticity index of E1, E2, and E3 emulsions under different storage times.

**Emulsion**	**SA**	**EI (×10** ^**−3**^ **, nm** ^**−2**^ **)**						
	**concentration (%)**	**D0**	**D14**	**D28**	**D42**	**D56**	**D70**	**D84**
E1	1	3.531 ± 0.353^f,E^	3.917 ± 0.167^e,E^	4.647 ± 0.096^d,H^	6.156 ± 0.190^c,G^	6.715 ± 0.092^b,G^	6.513 ± 0.115^b,I^	7.379 ± 0.086^a,D^
	1.25	4.872 ± 0.361^g,C^	5.697 ± 0.147^f,B^	7.461 ± 0.208^e,D^	10.270 ± 0.189^d,B^	10.751 ± 0.267^c,C^	11.419 ± 0.152^b,D^	12.238 ± 0.193^a,B^
	1.5	5.267 ± 0.384^f,B^	5.520 ± 0.236^f,C^	8.459 ± 0.196^e,C^	9.098 ± 0.217^d,C^	10.362 ± 0.153^c,D^	12.587 ± 0.209^b,B^	23.458 ± 0.277^a,A^
E2	1	3.722 ± 0.294^f,D^	3.752 ± 0.180^f,F^	6.535 ± 0.138^d,E^	7.158 ± 0.174^c,F^	7.589 ± 0.154^ab,F^	7.653 ± 0.122^a,H^	7.406 ± 0.125^b,D^
	1.25	5.417 ± 0.401^e,B^	7.452 ± 0.066^d,A^	9.394 ± 0.081^c,B^	12.007 ± 0.278^b,A^	12.780 ± 0.197^a,A^	11.825 ± 0.335^b,A^	11.894 ± 0.326^b,B^
	1.5	6.140 ± 0.476^e,A^	4.246 ± 0.177^f,D^	10.341 ± 0.049^c,A^	8.026 ± 0.297^d,D^	12.150 ± 0.204^a,B^	12.229 ± 0.210^a,C^	11.346 ± 0.287^b,C^
E3	1	2.083 ± 0.039^g,H^	2.269 ± 0.182^f,I^	4.264 ± 0.101^d,I^	4.947 ± 0.132^c,H^	5.659 ± 0.187^b,H^	7.789 ± 0.067^a,G^	3.092 ± 0.058^e,G^
	1.25	2.899 ± 0.261^e,G^	2.592 ± 0.164^f,H^	6.106 ± 0.203^c,F^	7.433 ± 0.134^b,E^	7.497 ± 0.169^b,F^	9.776 ± 0.124^a,E^	3.849 ± 0.303^d,F^
	1.5	3.136 ± 0.274^f,F^	2.882 ± 0.139^g,G^	5.425 ± 0.080^e,G^	7.120 ± 0.163^c,F^	8.369 ± 0.181^a,E^	8.167 ± 0.164^b,F^	5.976 ± 0.047^d,E^

*D0, D14, D28, D42, D56, D70, and D84 represent different storage times of emulsions. Values with different lowercase letters represent significant difference within the line (P < 0.05). Values with different capital letters represent significant difference within the column (P < 0.05)*.

The MVI represents the macroscopic viscosity of the sample. As shown in Table 4, the MVI of the fresh emulsions of E1, E2, and E3 has increased with the increase of the SA concentration, indicating that SA influenced the viscosity characteristics of the emulsion system. The MVI of emulsions has increased to different degrees, and thereunto, the MVI of E1 and E2 emulsions has a more visible increase than that of the E3 emulsions. This might be due to the enhancement of the three-dimensional network structure of the emulsion leading to the obstruction of the droplet movement, which was macroscopically manifested as the viscosity of the emulsion system increased.

**Table 4:** Macroscopic viscosity index of E1, E2, and E3 emulsions under different storage times.

**Emulsion**	**SA**	**MVI (×10** ^**−2**^ **, nm** ^**−2**^ **,s)**						
	**concentration (%)**	**D0**	**D14**	**D28**	**D42**	**D56**	**D70**	**D84**
E1	1	0.360 ± 0.144^f,D^	0.466 ± 0.080^f,C^	1.690 ± 0.331^e,E^	10.280 ± 0.771^d,C^	19.894 ± 0.808^b,A^	16.626 ± 0.974^c,C^	21.440 ± 4.171^a,D^
	1.25	0.386 ± 0.087^e,D^	0.544 ± 0.061^e,B^	3.392 ± 0.950^d,D^	16.511 ± 0.387^c,A^	15.733 ± 0.879^c,B^	19.22 ± 0.86^b,B^	26.99 ± 6.31^a,BC^
	1.5	0.461 ± 0.108^e,C^	0.457 ± 0.042^e,C^	7.912 ± 0.559^d,C^	8.614 ± 0.564^d,D^	12.735 ± 0.626^c,C^	43.056 ± 2.770^b,A^	146.105 ± 3.604^a,A^
E2	1	0.475 ± 0.147^e,C^	0.310 ± 0.031^e,D^	3.712 ± 0.363^d,D^	13.849 ± 1.400^c,B^	19.534 ± 1.320^b,A^	13.253 ± 0.629^c,E^	23.784 ± 2.313^a,CD^
	1.25	0.642 ± 0.151^e,B^	1.230 ± 0.060^e,A^	23.127 ± 1.871^b,A^	16.892 ± 0.756^c,A^	9.807 ± 0.592^d,D^	15.912 ± 0.549^c,C^	29.751 ± 2.856^a,B^
	1.5	0.708 ± 0.124^f,A^	0.180 ± 0.008^g,E^	15.020 ± 0.179^a,B^	2.673 ± 0.433^e,EF^	8.820 ± 0.660^d,E^	14.568 ± 0.653^b,D^	10.522 ± 0.647^c,E^
E3	1	0.076 ± 0.011^f,F^	0.107 ± 0.038^f,F^	0.877 ± 0.147^d,F^	1.843 ± 0.240^a,G^	1.442 ± 0.120^b,G^	1.011 ± 0.115^c,G^	0.329 ± 0.018^e,F^
	1.25	0.160 ± 0.029^f,E^	0.095 ± 0.022^f,F^	0.474 ± 0.118^e,F^	3.333 ± 0.274^b,E^	4.100 ± 0.246^a,F^	1.060 ± 0.046^c,G^	0.752 ± 0.161^d,F^
	1.5	0.162 ± 0.022^f,E^	0.126 ± 0.011^f,F^	0.463 ± 0.092^e,F^	2.273 ± 0.132^b,FG^	3.856 ± 0.108^a,F^	2.207 ± 0.127^c,F^	1.165 ± 0.046^d,F^

*D0, D14, D28, D42, D56, D70, and D84 represent different storage times of emulsions. Values with different lowercase letters represent significant difference within the line (P < 0.05). Values with different capital letters represent significant difference within the column (P < 0.05)*.

In conclusion, SA of different M/G ratios had an important influence on the fluidity of the emulsion, which was manifested as the E3 emulsion system, prepared by SA-3 (with high G blocks), could reach the solid-liquid equilibrium state later and remain in a flow condition for a longer time in the storage. However, the E1 and E2 emulsions, prepared by SA-1 and SA-2 (with high M blocks), had stronger viscoelastic properties and tended to form gel structures that limit droplet movement.

### Effect of Different Water-Oil Ratios on Properties of Emulsion

Lim et al. found that the water-oil ratio affected the particle size of emulsion (38), as well as the particle size associated with its stability. The influence of water-oil ratio on the properties of the emulsion was further investigated by measuring the particle size, CI, microstructure, rheological, and microrheological properties of the emulsion with different water-oil ratios. As previously described, E3 emulsion had a good fluidity while maintaining stability. Therefore, O1 and O2 emulsions, with 20:1 and 10:1 water-oil ratios, were prepared by SA-3 and CaCO_3_-8, respectively.

**Figure 7:**
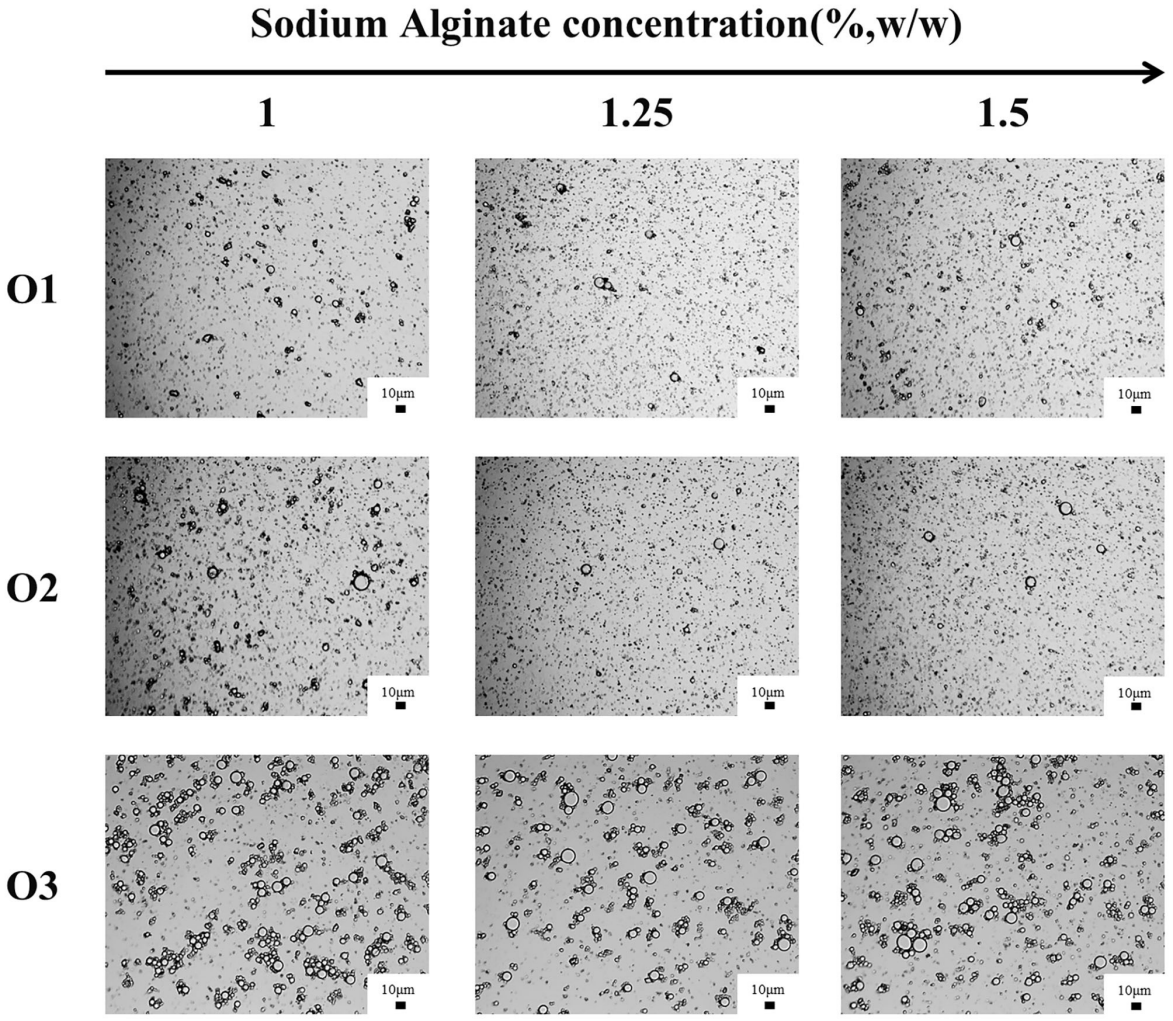
Optical microstructure images of O1, O2, and O3 emulsions stored for 28 days (scale bar is 10 μm).

Table 5 showed the droplet sizes and the CI (after stored for 2 h) of emulsions with different volumes of oil phase. The particle size of O1, O2, and E3 emulsions increased, while its CI has decreased gradually when the water-oil ratios decreased from 20:1 to 5:1. It was also confirmed by the microscope image that the particle size of emulsion increased with the decrease of the water-oil ratio (Figure 7). The reason might be that the increase of the volume of oil phase has increased the oil-water interface in the emulsion system, causing the surface of droplets to be insufficiently covered by CaCO_3_ and also by SA in the shear process. Thus, droplets of emulsion were gathered to form larger droplets to reduce the oil-water interface and to form a stable coverage. Studies have shown that excessive droplet aggregation would cause emulsion stratification and sediment, reduce emulsifying efficiency, and affect the stability of emulsion (39, 40). Moreover, the particle size and the CI of the emulsion have decreased with the increase of the SA concentration (Table 5), verifying that the increase of SA concentration was conducive to improving the stability of the emulsion.

**Table 5:** Particle size and creaming index of emulsions with different oil phase volumes.

**SA concentration (%, w/w)**	**O1**		**O2**		**O3**	
	**D[4, 3] (μm)**	**CI (%)**	**D[4, 3] (μm)**	**CI (%)**	**D[4, 3] (μm)**	**CI (%)**
1	4.59 ± 0.08	87.70	6.30 ± 0.33	82.71	8.57 ± 0.14	59.42
1.25	4.28 ± 0.02	85.88	5.59 ± 0.32	81.59	7.33 ± 0.03	44.22
1.5	4.04 ± 0.05	84.70	4.90 ± 0.07	79.03	6.52 ± 0.14	30.11

*D[4, 3] represents the volume mean diameter and CI represents the creaming index*.

**Figure 8:**
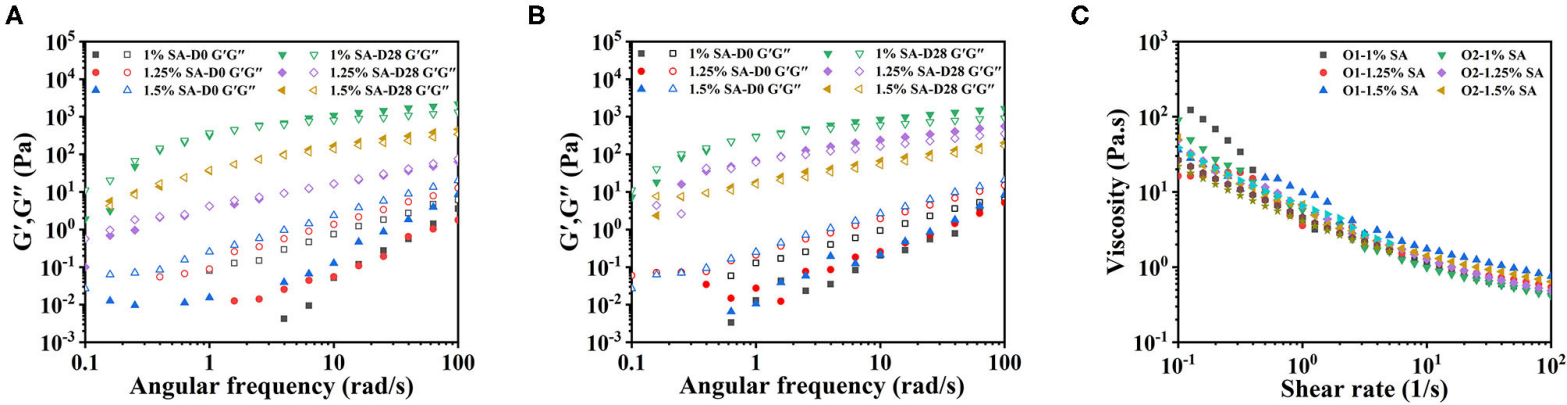
Rheological properties of O1 and O2 emulsions: dynamic frequency sweep images of O1 **(A)** and O2 **(B)** emulsions. and steady-state shear sweep images of the emulsions stored for 28 **(C)** days. D0 and D28 represent emulsions stored for 0 and 28 days.

Figure 8 showed the dynamic frequency sweep and the steady-state shear sweep results of the emulsion system as measured by the rotational rheometer at 0 and 28 days of storage. The G” of O1, O2, and E3's fresh emulsions was greater than G'. The G” was basically equal to G' after 28 days of storage, and the G” and G' increased with the extension of the storage time (Figures 8A,B). These meant that the elastic structure of the emulsion system was constantly generated, and the network structure was enhanced. The O1, O2, and E3 emulsions had obvious thinning behavior after 28 days of storage (Figure 8C), representing that they were all non-Newtonian fluids.

**Figure 9:**
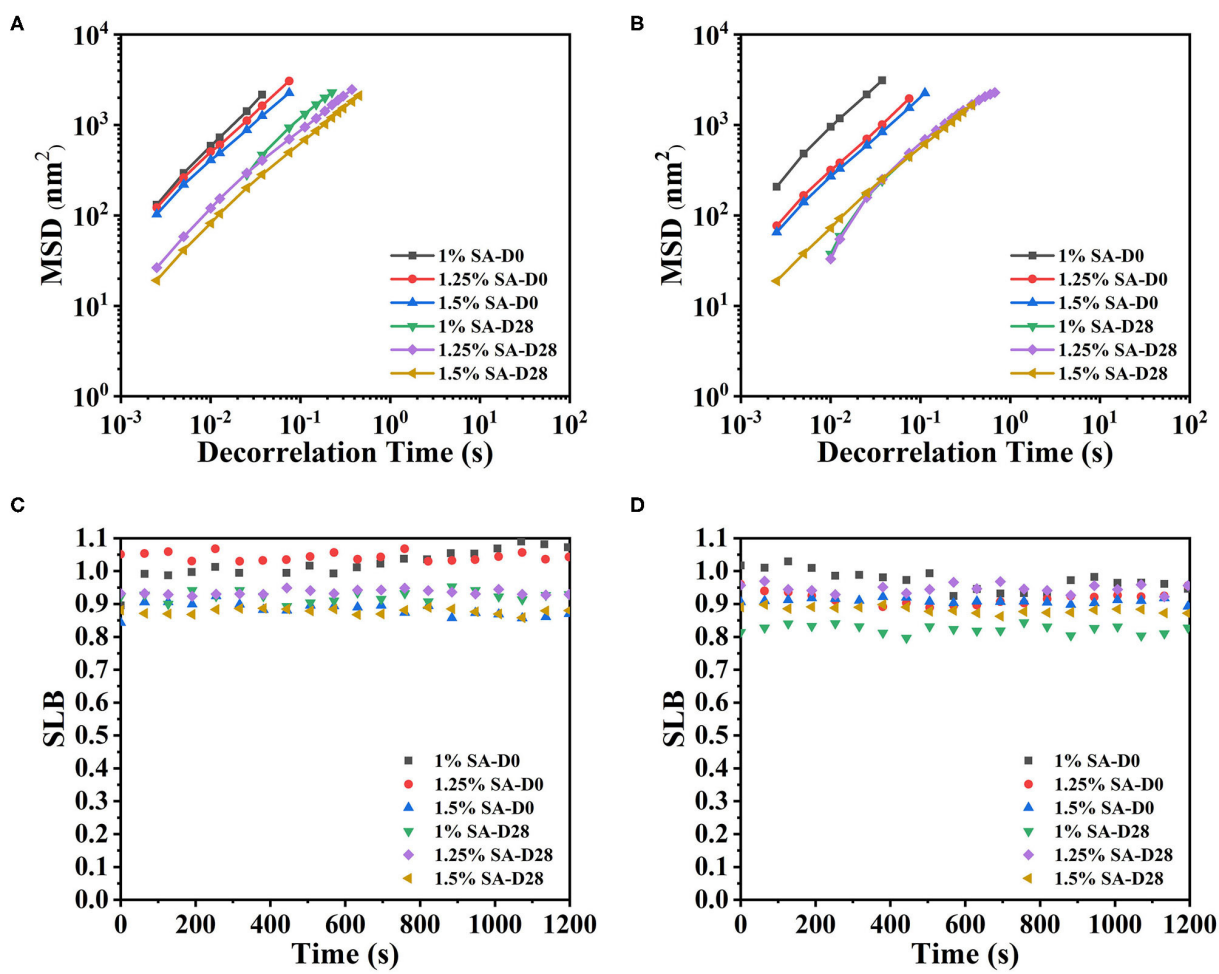
MSD curves and SLB of O1 **(A,C)**, and O2 **(B,D)** emulsions. D0 and D28 represent emulsions stored for 0 and 28 days.

The microrheological properties of O1, O2, and E3 emulsions on day 0 and 28 were shown in Figure 9. It could be seen clearly that the MSD curves of O1, O2, and E3 emulsions on day 0 and 28 were linear curves. The SLB of emulsions exhibited a downtrend with the storage time but were always greater than 0.5. These demonstrated that the droplets movement of O1, O2 and E3 emulsions was relatively free and the viscous properties were dominant throughout the 28 days. In contrast, the E3 emulsion had a longer decorrelation time and smaller SLB, suggesting that the E3 emulsion was closer to a solid-liquid equilibrium state, which is, potentially, because the increase of oil phase volume has also increased the viscosity of the emulsion system and has limited the Brownian motion of the droplet.

In summary, the oil phase, as a critical component of the emulsion system, had a certain effect on the physical properties of emulsion. Specifically, the emulsion with a larger oil phase volume had a larger particle size and a stronger viscoelasticity.

### Calcium Release Properties of Emulsion-Based Calcium Supplements

As previously mentioned, as this experiment prepared the emulsion stabilized by CaCO_3_ and SA, a new type of gastric floating calcium supplement was constructed based on the development status of calcium supplements. By simulating stomach digestive conditions *in vitro*, the calcium release behavior of emulsions with different M/G ratios of SA and different water-oil ratios in SGF was studied. The reasons affecting the release of Ca^2+^ in the emulsions were also analyzed to further optimize the emulsion system.

**Figure 10:**
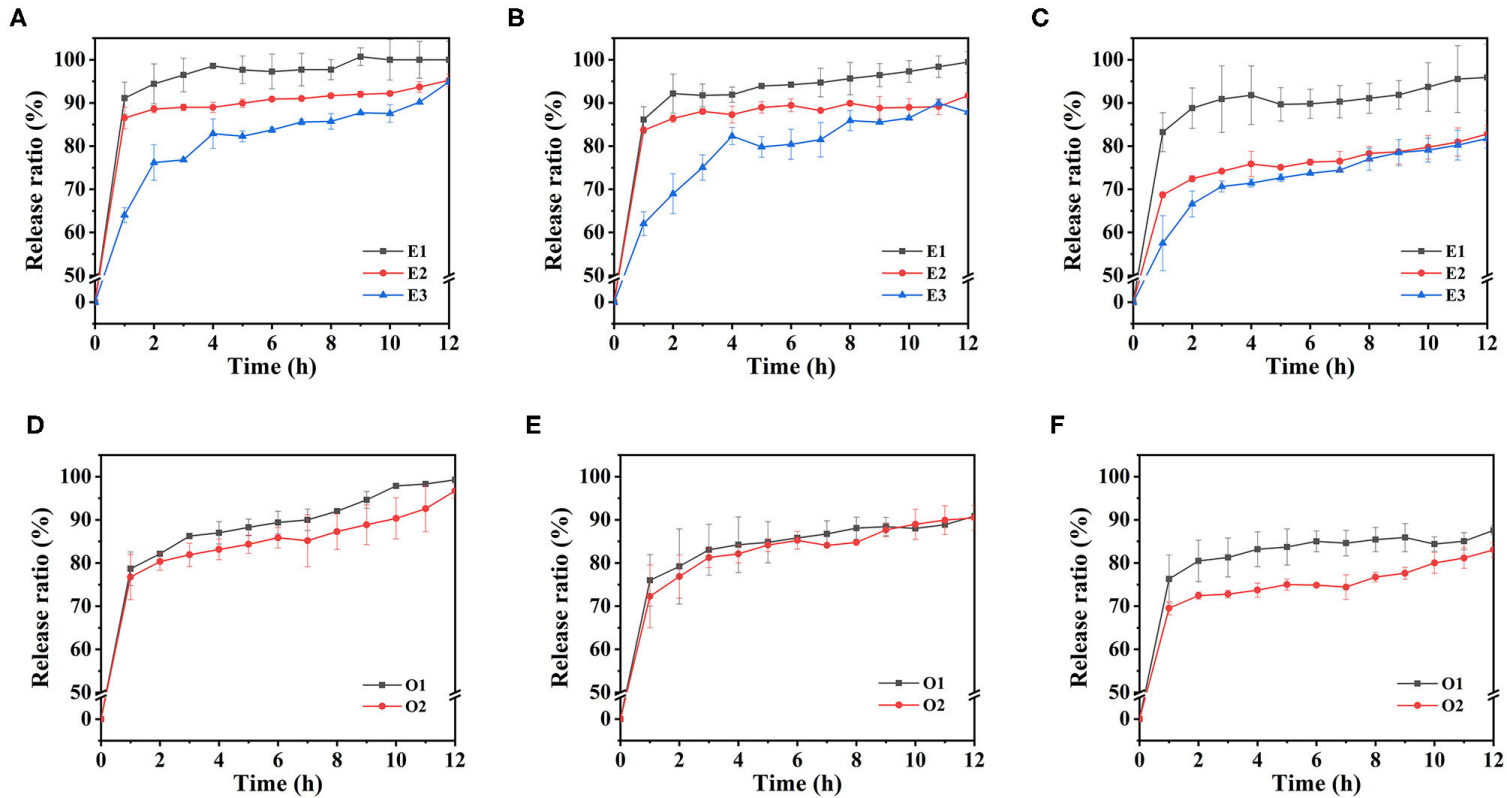
The calcium release curves of emulsions in simulated gastric fluid environment: E1, E2, and E3 emulsions with 1% SA **(A)**, 1.25% SA **(B)** and 1.5% SA **(C)**, O1 and O2 emulsions with 1% SA **(D)**, 1.25% SA **(E)** and 1.5% SA **(F)**.

The calcium release curves of the E1, E2, and E3 emulsion-based calcium supplements in SGF were shown in Figures 10A–C. All the emulsion systems containing the different concentrations of sodium alginate have shown a release lasting for 12 h in a simulated stomach environment. The release process can be divided into two stages accordingly, the first stage was the rapid release of calcium in 0-1 h, while the second one was the slow release of calcium in 1-12 h. The reason for this phenomenon was that CaCO_3_ rapidly decomposed into Ca^2+^ when the emulsion was added to SGF. Part of the Ca^2+^ was quickly released into the SGF, and the other part of Ca^2+^ combined with the uronic acid fragment of SA to form a gel structure in the outer layer of the emulsion, which prevented the direct contact between the remaining CaCO_3_ particles and SGF.

The calcium release behavior of E1, E2, and E3 emulsions in SGF existed with differences. Under the condition of 1% SA concentration, the calcium release ratio of E1 emulsion reached 91.12% in 1 h, and then entered the slow-release stage, reaching 100% in 9 h. The calcium release ratio of E2 emulsion reached 86.52% in 1 h and 95.23% in the end. The calcium release ratio of E3 emulsion reached 64.01% in 1 h, and the final cumulative release was 94.92%. It could be clearly found that E1 emulsion had the highest calcium release ratio, followed by E2 emulsion and E3 emulsion. This might be because the SA with a higher G-block ratio would form a stronger gel structure when crosslinking with Ca^2+^, thus, maintaining the integrity of the gel morphology for a longer time and having a lower Ca^2+^ release ratio. The results suggested that M/G ratio affects the calcium release of the calcium supplements in the stomach by influencing the gel structure of SA, which was shown as the cumulative calcium release ratio of the emulsion-based calcium supplements that were also increased with the increase of M/G ratio. According to this feature, different calcium release requirements can be satisfied. Furthermore, the calcium release ratio of the emulsion has decreased with the increase of the SA concentration. This was due to the fact that there were more binding sites for gel formation when SA concentration was high; more Ca^2+^ was used for gel formation and the release was slowed down.

The calcium release curves of emulsions with different oil phase volumes in SGF were shown in Figures 10D–F. When the SA concentration was 1%, the calcium release ratio of O1 emulsion reached 78.69% in 1 h, continued to release for 12 h, and the final cumulative release was 99.28%. The final release ratios of O2 and E3 emulsions were 96.73 and 94.92%, respectively. It could be found that the calcium release ratio decreased with the increase of the volume of oil phase in the emulsion system. This might be due to the increase of the oil phase volume, which increased the particle size of the emulsion, reduced the oil-water interface area, and subsequently reduced the contact area between the emulsion system and the SGF. Therefore, the Ca^2+^ that was dissolved from the CaCO_3_ was correspondingly lesser, which was reflected in the decrease of the cumulative release ratio of calcium.

## Conclusions

The primary focus of this study is on the effects of the different M/G ratios of the SA on the physicochemical properties of the emulsion stabilized by CaCO_3_ and SA. The results showed that the smaller M/G ratio of SA could prepare the emulsion with a smaller particle size. After 6 months of storage, E1 and E2 emulsions, prepared by SA with a higher M/G ratio, showed gelation behavior, while E3 emulsion, prepared by SA with a lower M/G ratio, remained in flow condition. Rheological and microrheological results demonstrated that E1 and E2 emulsions had stronger viscoelastic properties and tended to form gel structures that limited the droplet movement, while E3 emulsion was dominated by viscous behavior. Additionally, the emulsion with larger oil phase volume had a larger particle size and a stronger viscoelasticity. The study on the calcium release behavior of the emulsion indicated that the calcium release ratio increased with the decrease of SA concentration, with the increase of M/G ratio, and with the decrease of oil phase volume. To sum up, the difference of the M/G ratios of SA and water-oil ratios had a certain influence on the physicochemical properties of the emulsion system, subsequently causing the difference in calcium release characteristics of the emulsion, which could be used to exploit the emulsion-based calcium supplements with the demands of different calcium release.

## Data Availability Statement

The raw data supporting the conclusions of this article will be made available by the authors, without undue reservation.

## Author Contributions

JL and BL: conceptualization and supervision and funding acquisition. XY, HS, HL, and JL: methodology. JL, HS, and XY: investigation and data curation. XY, HS, and HL: formal analysis. XY and JL: writing—original draft. JL, XY, and HL: writing—review and editing. JL: critical revision of the manuscript. All authors contributed to the article and approved the submitted version.

## Funding

This work was supported by the Hubei Provincial Natural Science Foundation for Innovative Group (grant no. 2019CFA011).

## Conflict of Interest

The authors declare that the research was conducted in the absence of any commercial or financial relationships that could be construed as a potential conflict of interest.

## Publisher's Note

All claims expressed in this article are solely those of the authors and do not necessarily represent those of their affiliated organizations, or those of the publisher, the editors and the reviewers. Any product that may be evaluated in this article, or claim that may be made by its manufacturer, is not guaranteed or endorsed by the publisher.
